# Human ATG3 binding to lipid bilayers: role of lipid geometry, and electric charge

**DOI:** 10.1038/s41598-017-15057-6

**Published:** 2017-11-15

**Authors:** Javier H. Hervás, Ane Landajuela, Zuriñe Antón, Anna V. Shnyrova, Felix M. Goñi, Alicia Alonso

**Affiliations:** Instituto Biofisika (CSIC, UPV/EHU) and Departamento de Bioquímica y Biología Molecular, Universidad del País Vasco, Bilbao, Spain

## Abstract

Specific protein-lipid interactions lead to a gradual recruitment of AuTophaGy-related (ATG) proteins to the nascent membrane during autophagosome (AP) formation. ATG3, a key protein in the movement of LC3 towards the isolation membrane, has been proposed to facilitate LC3/GABARAP lipidation in highly curved membranes. In this work we have performed a biophysical study of human ATG3 interaction with membranes containing phosphatidylethanolamine, phosphatidylcholine and anionic phospholipids. We have found that ATG3 interacts more strongly with negatively-charged phospholipid vesicles or nanotubes than with electrically neutral model membranes, cone-shaped anionic phospholipids (cardiolipin and phosphatidic acid) being particularly active in promoting binding. Moreover, an increase in membrane curvature facilitates ATG3 recruitment to membranes although addition of anionic lipid molecules makes the curvature factor relatively less important. The predicted N-terminus amphipathic α-helix of ATG3 would be responsible for membrane curvature detection, the positive residues Lys 9 and 11 being essential in the recognition of phospholipid negative moieties. We have also observed membrane aggregation induced by ATG3 *in vitro*, which could point to a more complex function of this protein in AP biogenesis. Moreover, *in vitro* GABARAP lipidation assays suggest that ATG3-membrane interaction could facilitate the lipidation of ATG8 homologues.

## Introduction

Macroautophagy is a bulk degradation pathway conserved among eukaryotic cells^1^. This process is characterized by the generation of a double membrane structure called autophagosome (AP) which engulfs organelles or cytoplasmic portions and subsequently delivers the material into the lysosome for degradation^2^. AP formation requires more than 30 autophagy-related (ATG) proteins acting in a hierarchical way^3^. Among these proteins an ubiquitin-like (UBL) system, composed by Atg7, Atg3 and Atg8, triggers the covalent attachment of Atg8 (LC3 and GABARAP subfamilies in mammals) to phosphatidylethanolamine (PE), a lipid found in the AP membrane^4,5^. It has been shown that Atg3 is responsible for transferring Atg8 to the membrane^6,7^, where Atg8 participates in AP formation, maturation and closure. In fact, it has been described that mammalian Atg8-like proteins (LC3, GATE-16 and GABARAP) induce membrane tethering and fusion *in vitro* ^8–10^, which could be related to the AP growth process. The correct equilibrium of these processes is vital for the maintenance of cell homeostasis, and their imbalance is related to human disorders e.g. neurodegenerative diseases and cancer.

Human ATG3, the E2-like enzyme for Atg8 conjugation, contains some disordered regions that allow its classification among the intrinsically disordered proteins^11^. This property is found in proteins that participate in processes required for quick cellular responses, such as autophagy. Atg3 handle region (HR) and flexible region (FR) have been found to interact with Atg7 and Atg8, respectively^12^. These two domains act together with the catalytic cysteine (Cys264) to catalyse ATG3-LC3 binding, upon which LC3 partitions to the membrane^13^. Atg3 N-terminus has been proposed to be the protein region responsible for membrane detection and interaction^14^. Specifically, the N-terminal amphipathic α-helix domain of the protein was shown to be a membrane-curvature sensing domain^15^. Recent data^16^ indicate that acetylation enhances Atg3 membrane binding and Atg8 lipidation. The details of Atg3 interaction with the membrane lipid matrix are, to our knowledge, virtually unexplored. Due to the ability of Atg3 to interact with Atg12^17,18^, Atg12-Atg5-Atg16L1 would promote a more efficient recruitment of Atg3 to membranes *in vivo* ^19,20^. Atg12-Atg5 induces a reorientation of Atg3 catalytic cysteine toward a threonine residue so that Atg3 conjugase activity is stimulated^13,21^. Apart from its specific role in autophagy, the possible implication of Atg3 in the regulation of mitochondrial homeostasis, cell death and mitophagy has also been proposed^17,22^.

Here we show that the N terminus of human ATG3 is involved in the binding of the protein to uncharged model membranes containing phosphatidylethanolamine, the degree of binding being directly proportional to membrane curvature. When negatively-charged lipid species are present in the membrane, ATG3 binding is favored in all types of membrane templates used in this study, by-passing the membrane curvature enhancement required for neutral membrane compositions. Additionally, we have observed the effect of membrane tethering induced by the ATG3 *in vitro*, which might point to a more complex role of this protein in the process of AP formation.

## Results

### ATG3 binding to lipid monolayers and bilayers

**Figure 1:**
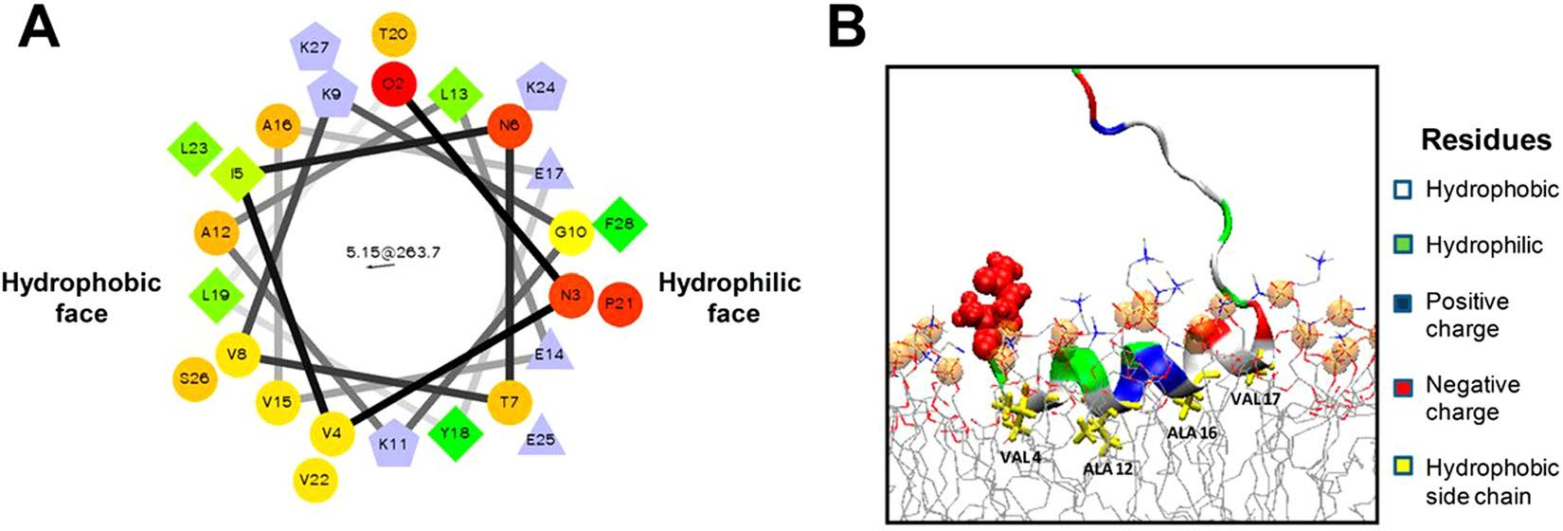
Predicted structure of ATG3 N-terminal amphipathic α-helix. (**A**) Helical wheel representation of the predicted N-terminal amphipathic helix (amino acids 2–27) of human ATG3 (wheel generated in Helical Wheel Projections). (**B**) The predicted insertion of ATG3 N-terminal α-helix into membranes. Hydrophobic (Leu, Ala, Val), negatively-charged (Glu) and positively-charged (Lys) residues are coloured yellow, red and blue respectively (courtesy of Dr. Martin B. Ulmschneider, Johns Hopkins University, Baltimore, MD). Peptide binding onto a lipid bilayer was studied by unbiased atomic detail molecular dynamics simulations following a protocol described in detail elsewhere^59^. In brief, the peptide was initially placed in bulk water in a fully extended conformation and allowed to freely fold and bind to the membrane. No biasing potentials were applied. The peptide was found to bind irreversibly and fold onto the interface within one microsecond.

ATG3 has been proposed to direct human Atg8 homologues (LC3, GATE-16 and GABARAP) to the membrane to complete their lipidation process. Some studies have demonstrated an important implication of the Atg3 N-terminal region in membrane recognition^14^. A predicted N-terminal amphipathic alpha-helix (Fig. 1), expected to be a membrane curvature sensor, would be responsible for this recognition^15^. In order to assess the direct implication of the N-terminal region of ATG3 in membrane binding and curvature sensing, we produced the ATG3 K9D/K11D mutant, in which the sequence predicted to fit into the bilayer-water interface is modified chemically and electrically.

**Figure 2:**
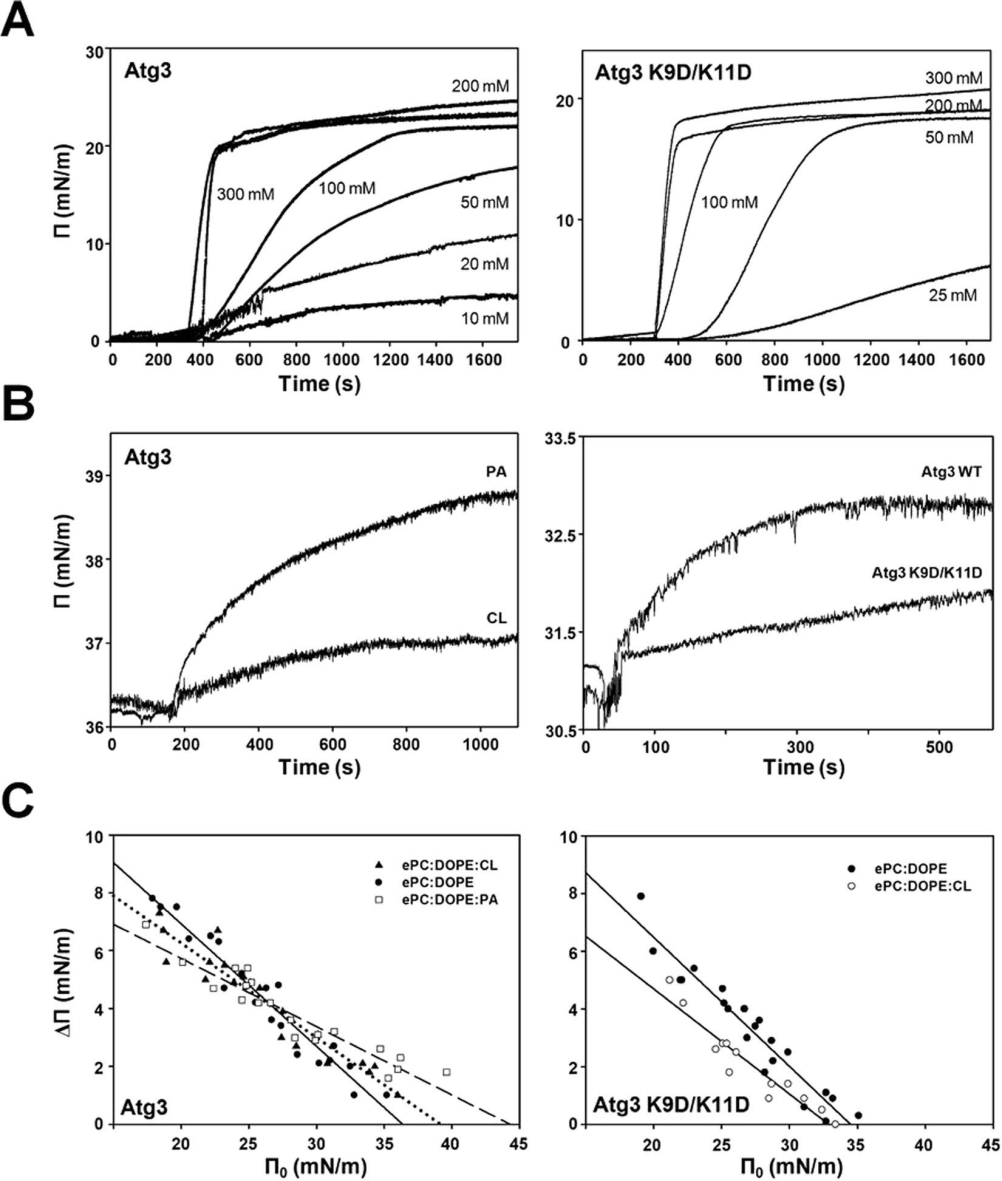
ATG3 and ATG3 K9D/K11D insertion into lipid monolayers. (**A**) Representative time courses of adsorption of ATG3 (left) or ATG3 K9D/K11D (right) at an air-water interface. (**B**) Representative time courses of increase in lateral pressure after ATG3 (0.2 μM) addition to PC:DOPE:CL (60:30:10 mol ratio) or PC:DOPE:PA (50:30:20 mol ratio) monolayers (left). Representative time courses of increase in lateral pressure after ATG3 or ATG3 K9D/K11D addition to PC:DOPE:CL (60:30:10 mol ratio) monolayers (right). (**C**) Maximum increase in lateral pressure after ATG3 (left) or ATG3 K9D/K11D (right) addition to lipid monolayers. Lipids were: [⚫] PC:DOPE (70:30 mol ratio), [▲] PC:DOPE:CL (60:30:10 mol ratio), [▫] PC:DOPE:PA (50:30:20 mol ratio). Data reported as a function of initial lateral pressure.

We first assayed the ability of ATG3 and its mutant to interact with and insert into lipid monolayers formed at the air-water interface in a Langmuir balance. Figure 2A shows that addition of either native ATG3 or of its K9D/K11D mutant to the aqueous phase causes a rapid, dose-dependent increase in surface pressure Π. Thus these are surface-active proteins, in principle susceptible to bind lipid monolayers and bilayers. The mutant appears to be somewhat less potent than the native ATG3, but in both cases the maximum change in Π appears to be close to 20 mN/m. The monolayers composed of PC:DOPE (70:30 mol ratio), PC:DOPE:CL (60:30:10 mol ratio) and PC:DOPE:PA (50:30:20 mol ratio) were used to test the affinity of ATG3 for membranes containing different anionic phospholipids. Lipid names are given in full in the “Materials” section. The protein (0.2 μM) was injected into the subphase and the increase in surface pressure was monitored in real time. The increase in surface pressure Π is interpreted as the protein interacting with the lipids in the monolayer and becoming embedded in it. Both ATG3 and the mutant were able to insert into these lipid monolayers extended at an initial surface pressure ≥23 mN/m (Fig. 2B), i.e. above the maximum change in surface pressure caused by adsorption of the proteins at the air-water interface (Fig. 2A).

ATG3 exhibited a higher affinity for monolayers containing PA than for those containing CL (Fig. 2B, left-hand panel). When the monolayer contained CL (Fig. 2B, right-hand panel) the K9D/K11D mutant showed a markedly lower affinity than the native ATG3. When the new equilibrium was attained, a further change in the lateral pressure at equilibrium (Δπ) was observed for all monolayer compositions. The protein-induced change in Δπ decreased linearly for both proteins with the increase in initial pressure (π_0_), i.e. protein insertion became hindered at higher initial lateral pressures. Above a certain π_0_ value, protein insertion was no longer observed (Fig. 2C). Extrapolation of the Δπ versus π_0_ lines to Δπ = 0 provides the so-called critical surface pressure π_c_, above which no protein insertion occurs. π_c_ values shown in Table 1 for wild type and mutant ATG3 are close to or above the usually accepted average value of lateral pressure in the cell membranes (i.e.∼30–35 mN/m^23^). Interestingly, wild type ATG3 caused a larger surface pressure increase in PA-containing monolayers than in CL-containing monolayers, the net electrical charge being similar in both cases (Fig. 2B left-hand panel). However, mutation of the N-terminus lysine residues of the protein canceled its selectivity for anionic phospholipids (Fig. 2C right-hand panel). Thus the lysine residues close to the membrane interface might influence ATG3 insertion into the lipid matrix.

**Table 1:** Critical surface pressures (π_c_) of monolayers with different lipid compositions in the presence of ATG3.

Protein	ePC:DOPE (70:30)	ePC:DOPE:CL (60:30:10)	ePC:DOPE:PA (50:30:20)
ATG3	36.4 mN/m (±1.23)	39.2 mN/m (±1.67)	44.3 mN/m (±1.94)
ATG3 ^K9D/K11D^	34.5 mN/m (±0.81)	32.9 mN/m (±1.29)	

Data derived from experiments as shown in Fig. 2C. The tendency line-associated standard error is given in parenthesis for each π_c_.

**Figure 3:**
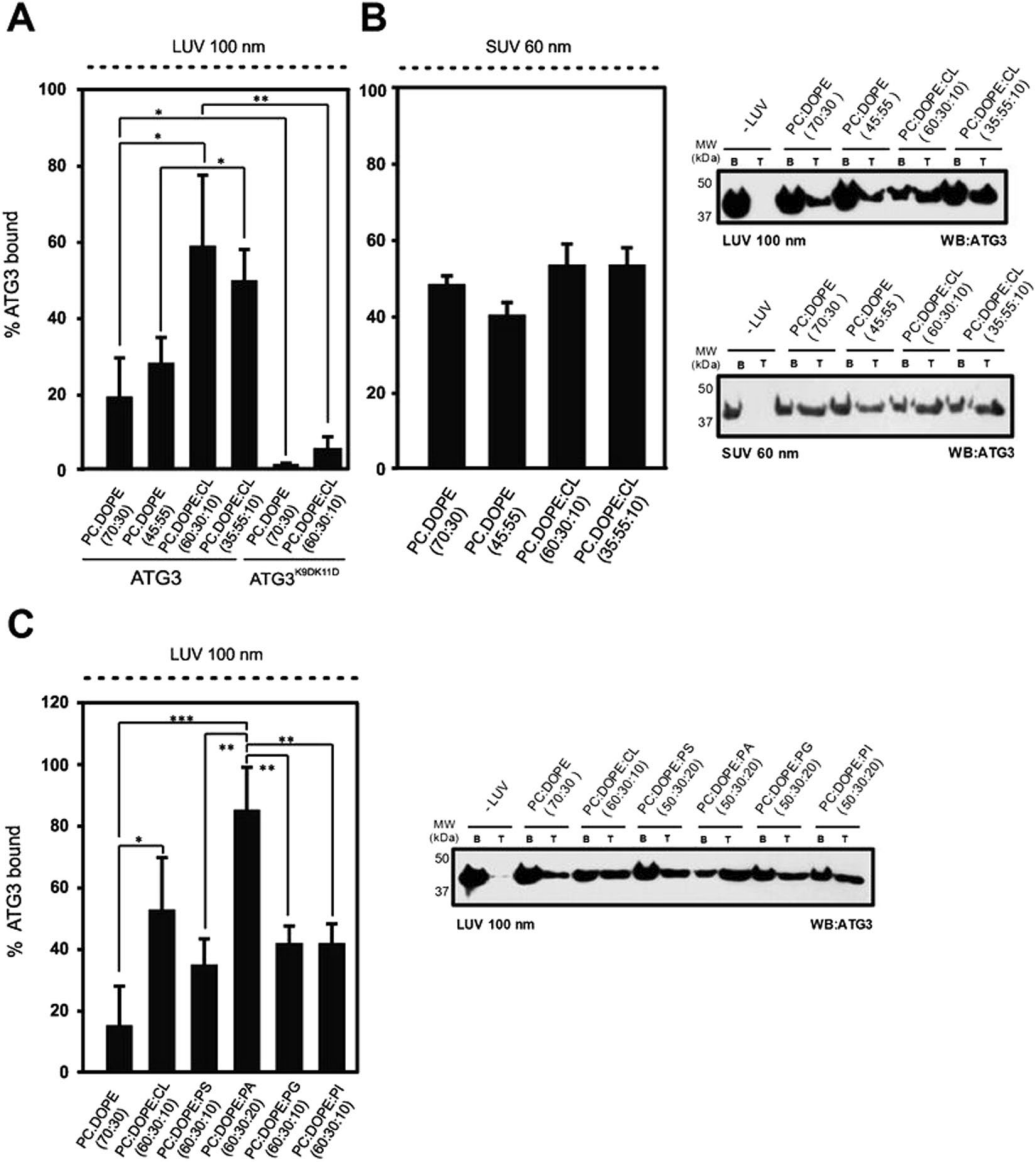
ATG3 interacts with negatively-charged phospholipid vesicles. Results from the flotation assay and quantification of the liposome-bound protein fraction of (**A**) 10 µM ATG3 or ATG3 K9D/K11D incubated with 3 mM LUVs (100 nm average size) (**B**) 10 μM ATG3 incubated with 3 mM SUVs (60 nm average size) (**C**) 10 μM ATG3 with LUV containing different anionic phospholipids. The gels show representative results. The data correspond to mean ± SEM (n = 3); **P = 0.001–0.01, ***P < 0.001. Full-length blots are presented in Supplementary Figure 3.

Next we explored whether these compositional preferences of the protein are also exhibited in the presence of a lipid membrane. Liposomal flotation assays were performed so that the composition and curvature that cell membranes would adopt *in vivo* could be mimicked. A fixed concentration of pure ATG3 (10 μM) was incubated with liposomes (1:300 protein:lipid mol ratio) and the mixture was subjected to an equilibrium sucrose gradient centrifugation, in which the vesicles have the capacity to float up, and the amount of protein interacting with these liposomes can be quantified. According to previous studies with a UBL system, 55% DOPE was required for an optimal lipidation of LC3, and also for aggregation and fusion of lipid vesicles^24^. As ATG3 is thought to be responsible for bringing over Atg8 homologues to the membrane, the preference of this protein for membranes with different amounts of DOPE and the influence of negatively-charged phospholipids on this interaction were tested. We found that ATG3 binds to DOPE-containing 100-nm LUVs, but no significant difference was observed between 30% and 55% DOPE (Fig. 3A). Moreover, when an anionic phospholipid (10% CL) was included into the LUV composition, the affinity of ATG3 for the membrane increased significantly (Fig. 3A). In the presence of CL no difference between 30% and 55% of DOPE was observed either. ATG3 K9D/K11D was not able to bind vesicles of any composition tested (Fig. 3A), this would be an example of a protein that can insert in a monolayer, but not in a bilayer. In general monolayer insertion is a requisite for bilayer insertion, but not all proteins that bind/insert into lipid monolayers are then able to insert into bilayers. These results suggest that ATG3 has a marked preference for negatively-charged membranes, and indicate that its N-terminal positively-charged residues play an important role in this interaction.

Melia and coworkers^15^ showed that mouse ATG3 contains a predicted N-terminal amphipathic helix that could be a membrane curvature sensor. In order to test whether the human ATG3 possesses this property, we compared the ATG3 binding to lipid vesicles of different sizes. As expected, decrease in membrane radius resulted in an increase of bound protein to neutral membranes. Indeed we observed an increase in the binding of ATG3 to PC/DOPE-containing SUV (60 nm in diameter) as compared to LUV (100 nm in diameter) (Fig. 3B). However with vesicles containing anionic phospholipids no ATG3 curvature preference was detected (Fig. 3B). Hence, ATG3 appears to behave as a membrane curvature sensor with neutral membranes, however the incorporation of negatively-charged phospholipids helps to by-pass this curvature effect.

Negatively-charged phospholipids are present in most cell membranes conferring some specific properties to the respective membrane compartments and playing a critical role in cell signaling. Since the origin of the autophagosomal membrane is still unknown, the preference of ATG3 for different anionic phospholipids was assessed using liposome flotation experiments. ATG3 showed a strong affinity for PA and CL, although its binding to liposomes containing PI, PG or PS was also significantly higher than to electrically neutral membranes (Fig. 3C). Considering that PA and CL, which gave the highest binding affinities, are “conically shaped” phospholipids, i.e. lipids with a negative intrinsic curvature^25^, we could suggest two factors that facilitate ATG3 interaction with membranes, namely lipids with an intrinsic negative curvature and net negative charges in the membrane. Aerolysin^26^, adenylate cyclase toxin^27^ or CTP:phosphocholine cytidyltransferase^28^ interactions with membranes are favoured by the incorporation of this kind of lipids, and this appears also to be the case for ATG3.

To summarize, the above results suggest that human ATG3 interacts with membranes containing physiological concentrations of DOPE and binds preferentially to membranes containing non-bilayer anionic lipids with negative spontaneous curvatures. The presence of anionic phospholipids decreases or suppresses the preference for highly curved vesicles, whereas the anionic lipids with a negative intrinsic curvature clearly facilitate ATG3 interaction with membranes. Furthermore mutations of Lys residues 9 and 11 in the predicted N-terminal α-helix reveal the important role of these residues in protein binding to lipid vesicles. Note that the natural anionic lipids used in this study may carry rather different fatty acyl chains, PI and CL being usually more unsaturated than PA or PG, thus the fluidity factor cannot be obviated when analyzing in depth the results in Fig. 3, this is a matter that deserves further study.

### Membrane tethering by ATG3

The ATG7/ATG3/LC3 UBL system is involved in autophagosome elongation. In this process vesicle tethering and fusion are essential. It is usually understood that the full responsibility for the latter processes falls on LC3. The other components (ATG7 and ATG3) are considered to act only in the previous conjugation reactions. However, since ATG3 interacts with membranes this protein could in principle contribute, to some extent, to the aggregation and fusion of vesicles. This hypothesis was tested as detailed below.

**Figure 4:**
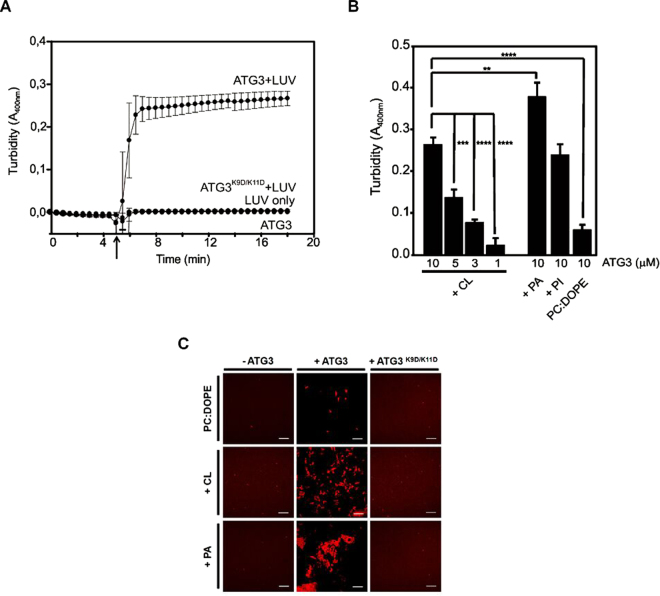
ATG3-promoted vesicle aggregation. (**A**) Time-course of 3 mM LUV (PC:DOPE:CL 60:30:10 mol ratio) aggregation induced by 10 μM ATG3. Arrow indicates addition of ATG3. Each curve represents the mean ± SE (n = 3). Note that data for ATG3, for LUV only and for ATG3 K9D/K11D + LUV overlap. (**B**) Effect of lipid composition and ATG3 concentration on LUV aggregation. Bars represent mean ± SE (n = 3) of ΔA400. ***P ≤ 0.001, **P ≤ 0.01, *P ≤ 0.05 of unpaired t-test. (**C**) Representative images of ATG3-induced aggregation of LUV of different lipid compositions: PC:DOPE (70:30 mol ratio), PC:DOPE:CL (60:30:10 mol ratio) or PC:DOPE:PA (50:30:20 mol ratio). Scale bars: 30 μm.

The ability of ATG3 to induce vesicle tethering was assessed by monitoring in a spectrophotometer the increase in sample turbidity (absorbance at 400 nm)^29^. ATG3 was observed to cause aggregation of electrically neutral vesicles [PC:DOPE (70:30 mol ratio)] and, at higher rates, of negatively-charged liposomes (containing PA, CL or PI) (Fig. 4A). Just as in the ATG3-vesicle interaction assay (Fig. 3) this protein showed the highest extent of tethering with PA-containing liposomes. In all cases, aggregation is ATG3 concentration-dependent, at least between 1 μM and 10 μM (Fig. 4A). Furthermore, the activity of ATG3 K9D/K11D was tested in parallel experiments and no vesicle aggregation was observed even with anionic vesicles (Fig. 4B). These results support the notion that the characteristics of ATG3-membrane association are directly related to this newly observed function, i.e. vesicle aggregation.

Confocal images of liposome aggregates confirmed the turbidity results. 10 μM ATG3 was incubated with liposomes of different lipid compositions, and after 1 h images were taken in a confocal microscope. Limited aggregation was observed with the PC:DOPE composition, while extensive liposome aggregation was seen with CL- or PA-containing vesicles (Fig. 4C). Our negative controls, buffer only or ATG3 K9D/K11D did not show any vesicle tethering.

Intervesicular lipid mixing and release of vesicle contents assays were performed in order to assess the possible contribution of ATG3 to the fusion processes in autophagosome elongation. However, ATG3 did not exhibit any of those activities under our experimental conditions (data not shown).

The above results illustrate a novel, easily testable activity for ATG3, namely vesicle aggregation. This activity would be in part dependent on the N-terminal α-helix as a membrane anchor, but other hydrophobic regions in the protein must also be involved (Fig. 2C). Our observations could suggest a possible contribution of ATG3 to vesicle tethering preceding fusion events in autophagosome elongation, while the fusogenic ability would remain in the final effector of the UBL system, Atg8. These aggregation and fusion processes would be facilitated in negatively-charged and highly curved membrane regions such as the edge of the isolation membrane, in which a relative enrichment of these proteins could exist.

### ATG3 Interaction with GUV containing negatively-charged lipids

Giant unilamellar vesicles (GUV) constitute a cell-sized model membrane system that allows direct visualization of particular membrane-related phenomena, such as domain formation or protein interaction, at the level of single vesicles using fluorescence microscopy-related techniques. This method was used to test whether or not ATG3 was able to interact with GUV of different lipid compositions, whether it had any lipid preference when binding to GUV, or whether we could reproduce the aggregation activity observed with highly curved membranes in this low-curvature system.

**Figure 5:**
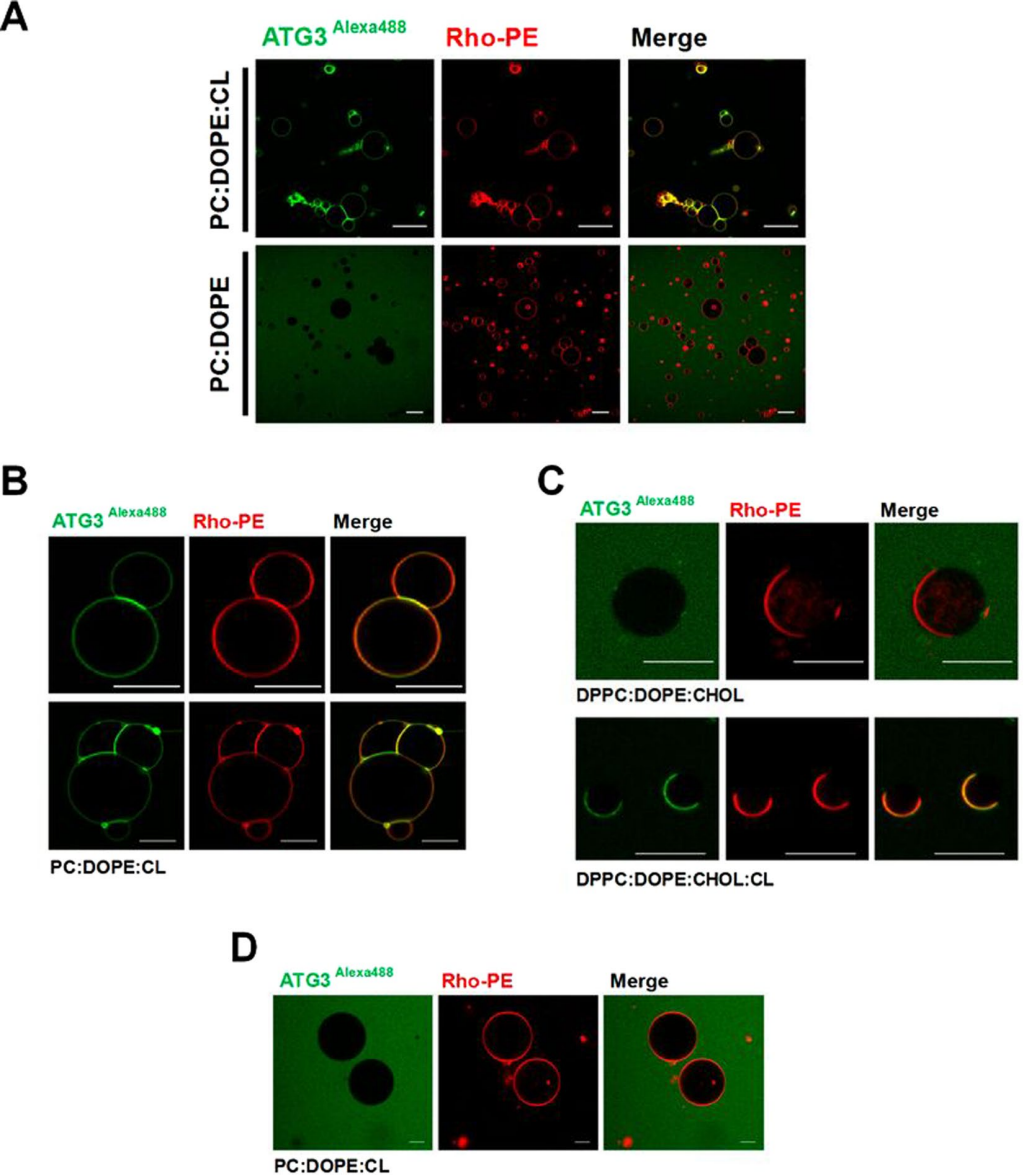
Interaction of ATG3 with cell-sized lipid vesicles, GUVs. (**A**) ATG3 interaction with GUV in the presence or absence of CL. Membranes labeled with Rho-PE and ATG3 labeled with Alexa 488. Merge: yellow indicates colocalization of both probes. Lipid composition is given at the left-hand side. Scale bars: 20 μm. (**B**) ATG3 preferential localization in GUV contact sites. (**C**) ATG3 binding to GUV with segregated domains. Two different lipid domains are clearly seen, one of them containing CL and labeled with Rho-PE. (**D**) ATG3 K9D/K11D does not interact with PC:DOPE:CL (60:30:10 mol ratio) GUV. Lipid compositions are given in each panel. Scale bars: 10 μm.

Alexa488-labeled ATG3 incubation with Rho-PE-containing GUV resulted in protein binding to GUVs containing negatively-charged phospholipids (Figs 5A and S1). We observed tethering of GUV membranes with preferential association of ATG3 to the GUV-GUV contact sites (Fig. 5B). No ATG3 binding was observed with electrically neutral GUV compositions (Fig. 5A).

To further analyze the preference of ATG3 for anionic phospholipids, GUV with segregated domains were electroformed. Asymmetric distributions of CL within the membrane of single vesicles were engineered by incorporating CL into lipid mixtures that are known to phase separate into two distinct liquid phases. Ternary mixtures of DOPE/DPPC/Chol have a large region of their phase diagram wherein these bilayer lipids segregate to form liquid-ordered (L_o_) and liquid-disordered (L_d_) domains^30^. The L_d_ phase of these model membranes is rich in DOPE due to its unsaturated hydrocarbon tails, and the L_o_ phase is rich in the saturated DPPC. Cholesterol partitions into both phases but is slightly enriched in the L_o_ domains as compared to the L_d_ phase. GUV composed of DOPE:DPPC:Chol (37.5:37.5:25 mol %) at 22 °C are within this region of liquid-liquid phase separation in the phase diagram. Domains of these two liquid phases coalesce in the individual vesicles to form two large domains on the vesicle surface, one of each phase. 10 mol% DOPE were exchanged for 10 mol% CL based upon two assumptions: that CL, due to its high degree of chain unsaturation, will preferentially partition into the L_d_ phase, and that despite such a significant change in lipid composition, the membrane will still be in a region of liquid-liquid phase coexistence^31^. ATG3-Alexa488 bound preferentially to regions of the membrane that are rich in CL (labeled with Rho-PE) (Fig. 5C). Thus, ATG3 interaction with GUV is determined by the presence of negatively-charged lipids on their surface. However, CL-containing GUVs were not labeled with ATG3 K9D/K11D-Alexa488. In fact, in the latter case the vesicles were seen as dark objects against a green (Alexa488) background (Fig. 5D)

**Figure 6:**
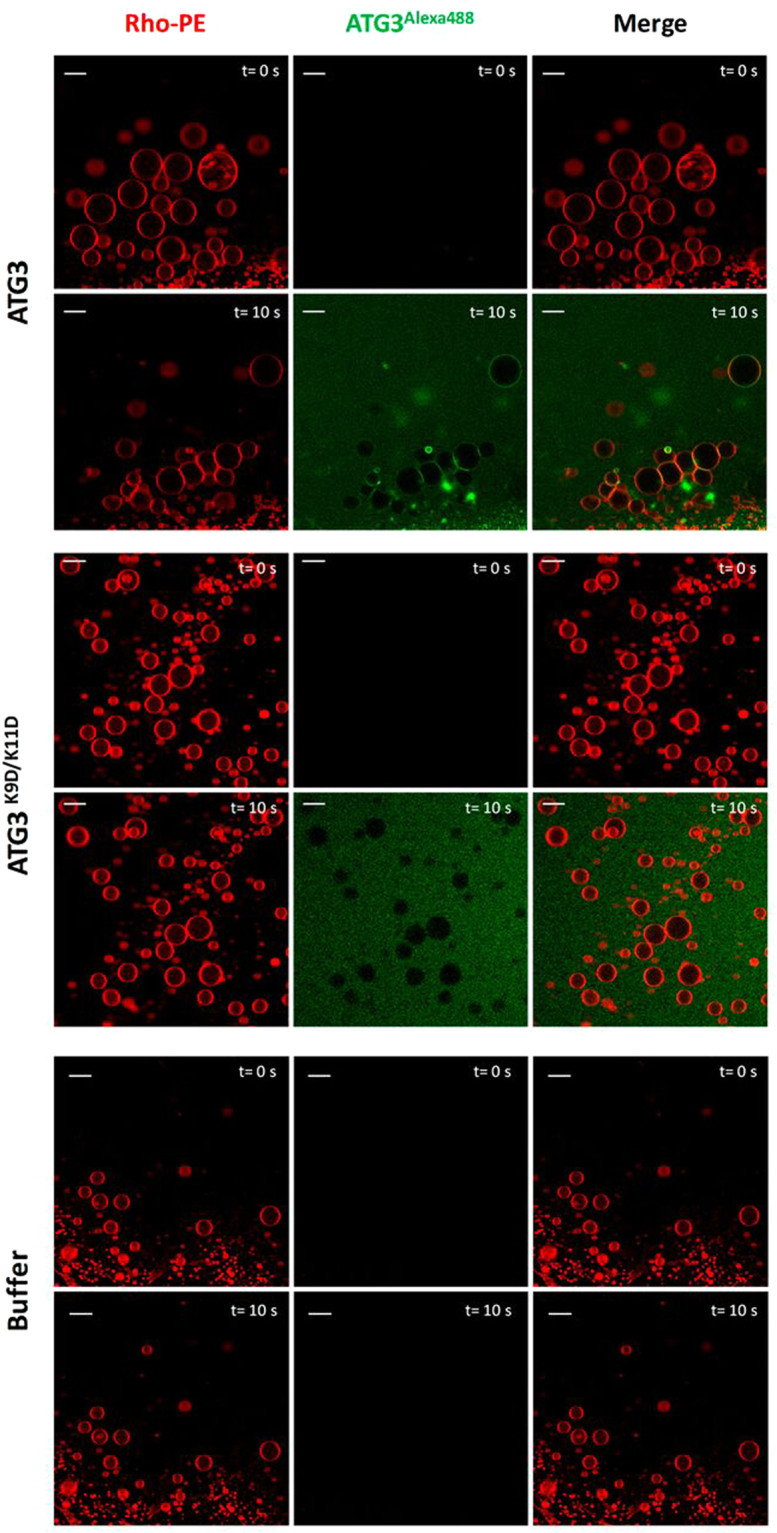
ATG3-induced GUV aggregation. Red channel corresponds to PC:DOPE:CL (60:30:10 mol ratio) GUVs containing 0.05 mol% of Rho-PE as membrane marker. Proteins (1 μM final concentration) were labeled with Alexa488 (green channel). 10 seconds after its addition to GUVs, ATG3 localized preferentially to vesicle contact sites. ATG3 K9D/K11D or buffer alone did not induce any effect on the GUVs.

In addition, ATG3 aggregation experiments could also be reproduced in GUV. A specific methodology was used to form GUV in order to obtain them close to each other to allow protein-induced tethering. In this case, PC:DOPE:CL (60:30:10 mol ratio) GUV were electroformed and directly visualized in a Nikon confocal microscope. Upon addition of ATG3-Alexa488 (1 μM) GUV tended to aggregate and precisely the flattened areas between tightly aggregated GUV appeared enriched in ATG3-Alexa488 (Fig. 6 upper panels). Controls made with ATG3 K9D/K11D-Alexa488 and buffer did not show any comparable event (Fig. 6 lower panels).

These results confirm that ATG3 interaction with anionic membranes does not require a high vesicle curvature (GUV curvature is practically zero). Instead, the intrinsic negative membrane curvature generated by cone-shaped lipid molecules and in part the electrostatic interaction with anionic phospholipids would be properties required for this specific association.

During autophagosome formation highly curved membrane regions emerge at the edge of this new structure. ATG8-driven fusion of new membrane material that allows PAS growth would occur in these regions. However, how the human ATG8 homologues are directed to the appropriate place to carry out their function is still under discussion. Hypotheses proposed by ours and other groups assume that ATG3 would be the effector for the detection of some special properties of a membrane region, such as curvature, structural defects or charges, where autophagosomal elongation would take place.

**Figure 7:**
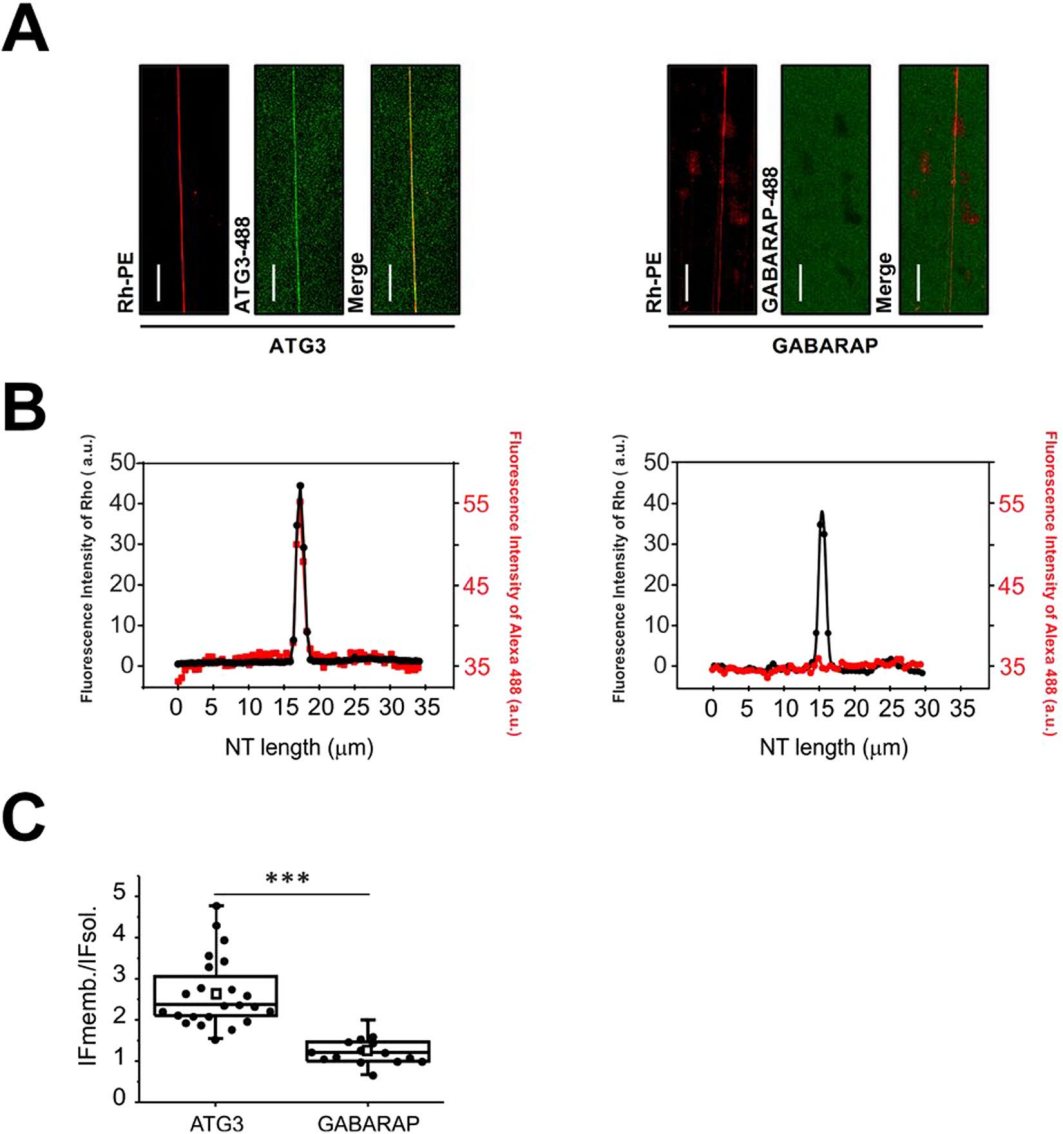
Binding of ATG3 (but not GABARAP) to neutral NTs. (**A**) ATG3 but not GABARAP (labeled with Alexa 488) binds to PC:DOPE (70:30 mol%) NTs containing 0.05 mol% of Rho-PE as membrane marker. Scale bars: 20 µm. (**B**) Fluorescence intensities of Rho-PE (black) and ATG3-A488/GABARAP-A488 (red) from the pictures in A were analyzed using the Image J function “Plot Profile”. Here the x-axis represents the horizontal distance through the selection and the y-axis the vertically averaged pixel intensity. For each condition, at least 20 nanotubes from 3 independent experiments were analyzed. (**C**) From the confocal fluorescence images, the ratio of mean integrated fluorescence intensity of ATG3 and GABARAP-488 in membrane (IFmemb.) and solution (IFsol.) was obtained from radial profiles. In the box-chart and raw data (dots) representation, squares represent mean of each condition, the “whisker” represents minimum and maximum values. The box plot goes from upper (75) percentile, median and lower (25) percentile of the values. For each condition, at least 20 nanotubes from 3 independent experiments were analyzed. ***p < 0.001.

As shown in the previous flotation studies with SUV, ATG3 increases its binding to neutral membranes when vesicle size decreases. To visualize this effect under the microscope we generated lipid nanotubes (NTs) starting from a compositionally well-defined unilamellar membrane system, such as the SUPER templates^32,33^. The formed NTs are thin tubules with high membrane curvature, reported to be a powerful tool to analyze curvature-dependent binding of proteins. ATG3-Alexa488 interacted with electrically neutral (PC:DOPE) NTs, while GABARAP did not exhibit any binding to NTs with similar curvature (Fig. 7). Thus, as observed above, a high membrane curvature is enough to induce ATG3 binding, even if the addition of anionic lipids relieves this requirement.

### ATG3 interaction with membranes enhances GABARAP lipidation

**Figure 8:**
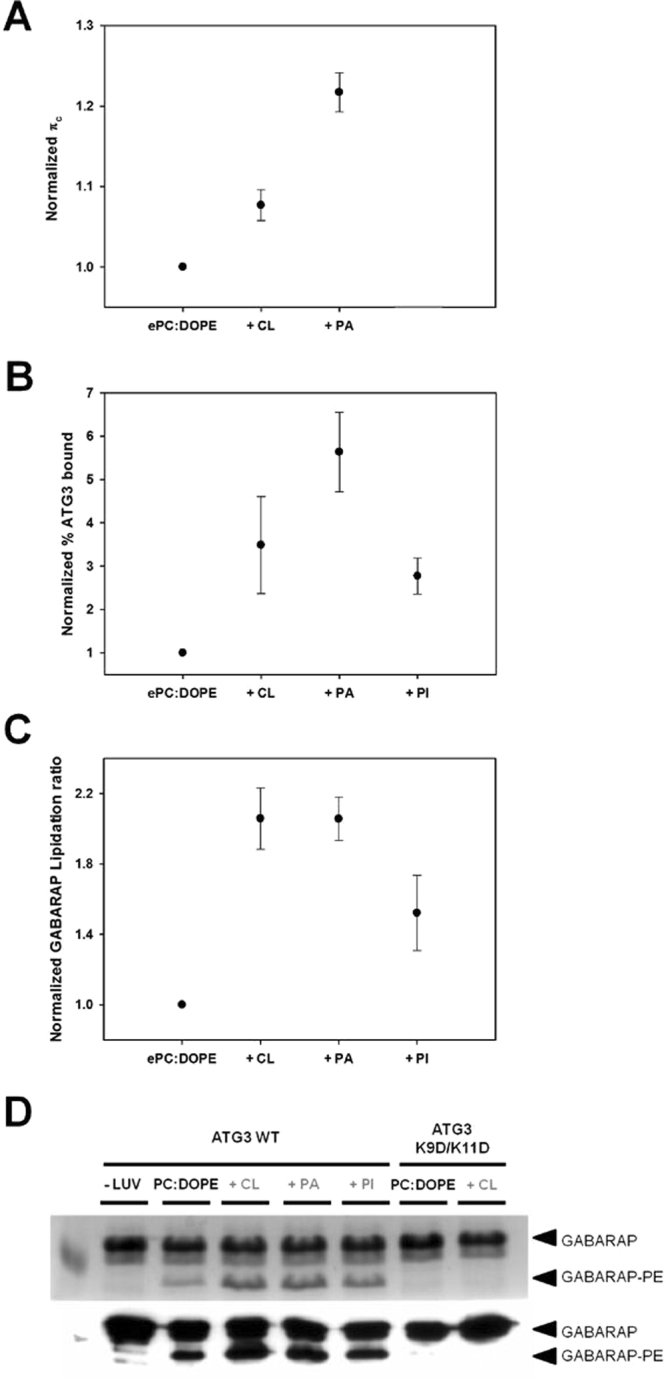
ATG3 governs GABARAP lipidation ratio. (**A**) ATG3 binding to lipid monolayers. Data from Fig. 2C normalized to the PC:DOPE value. (**B**) ATG3 binding to liposomes of different lipid compositions. Data from Fig. 3C normalized to PC:DOPE. (**C**) GABARAP lipidation ratio using different lipid compositions. Data obtained from gels as shown in D normalized to the PC:DOPE value. Average values of two experiments. (**D**) Coomassie blue staining (upper panel) and anti-GABARAP Western blotting (lower panel) of the *in vitro* lipidation reaction using different lipid compositions. Lipid compositions are detailed in each panel. Black arrow heads indicate lipidated and non-lipidated forms of GABARAP. Full-length blots/gels are presented in Supplementary Figure 3.

Atg3 was proposed to direct LC3 to the membrane for lipidation *in vitro* and, more recently, to act together with the Atg12-Atg5-Atg16L1 system to perform the same task *in vivo* ^20,34^. In this context, lipid composition changes could modify the lipidation rate of Atg8 homologues^4,15,35^. The effect of adding negatively-charged lipids, or lipids with a negative intrinsic curvature, that facilitate ATG3 binding to membranes, on GABARAP lipidation was analysed. For this purpose *in vitro* lipidation assays incubating ATG7, ATG3 and GABARAP in the presence of liposomes, ATP and MgCl_2_ were performed. The lipidation product was followed by SDS-PAGE gels and its subsequent Coomassie staining or Western blotting. GABARAP was used as the human Atg8 homologue in these experiments because the lipidated band of LC3 and GATE-16 was undistinguishable from the presumably adenylated form of the protein. The increased affinity of ATG3 for anionic monolayers (Fig. 8A) and vesicles (Fig. 8B) was also associated to an enhanced lipidation as observed in Fig. 8C,D. Moreover higher lipidation ratios were observed when lipids such as PA and CL, with a high negative intrinsic curvature, were incorporated to the liposomes. This suggests that ATG3 membrane interaction could govern the lipidation efficiency of the Atg8 homologues. This does not rule out a similar involvement of ATG7 in the regulation of Atg8 lipidation, for which no experimental data are available.

As an additional control the lipidation experiment in Fig. 8D was performed with GABARAP ΔG, and bilayers containing PC, DOPE, and either CL or PI. As expected, no lipidation was observed in any case (Fig. S2).

## Discussion

It is generally accepted that in AP elongation the protein Atg8 (and its mammalian homologues LC3, GATE-16 and GABARAP) triggers the aggregation and fusion of vesicles with the nascent autophagosome. Little is known of the way in which Atg8 is directed to the membrane. Some studies showed that Atg3 is involved in the transport and recognition of PE as an Atg8 substrate^36^, and also that another UBL system involved in autophagy, Atg12-Atg5-Atg16L1, interacts with membranes and improves the rate of Atg8 lipidation through interaction with Atg3^19^.

Atg3 was demonstrated to interact with membranes in part through its N-terminal region. It was also suggested that the predicted N-terminal amphipathic helix would be responsible for this attachment, and also that this helix would be a membrane curvature sensor^15^. Our results demonstrate the role of the N-terminal helix in ATG3-membrane interaction, and the particular affinity of ATG3 for negatively-charged membranes. In addition we have found that ATG3 induces aggregation of lipid vesicles, and enhances lipidation of Atg8 proteins.

### ATG3-membrane Interaction

The aim of this work was to obtain a better understanding of ATG3-membrane interaction. Using Langmuir balance measurements (Fig. 2) and liposome flotation assays (Fig. 3) we show that ATG3 binds PE-containing membranes and exhibits a particular affinity for negatively-charged membranes (Fig. 3A). Nath *et al*.^15^ indicated that ATG3 contains a membrane curvature sensor in its N terminus, but we show here that curvature recognition by ATG3 is only noticed with neutral membranes (Fig. 7). Indeed no change is observed in ATG3 binding when the size of anionic-lipid containing liposomes decreases (Fig. 3B). Furthermore, ATG3 attachment to membranes relies on electrostatic and hydrophobic interactions, since mutations in the N-terminal amphipathic helix, including polar and hydrophobic residues, alter ATG3 interaction efficiency^15^. Our results with the ATG3 K9D/K11D mutant (Fig. 3A) strongly support the idea that these two lysine residues, located between the hydrophobic and polar faces of the helix, constitute an important anchor for ATG3 binding, probably related to the increased binding capacity in the presence of anionic phospholipids. Moreover, comparing the different negatively-charged phospholipids we observe that lipid packaging defects in the membrane can be important for ATG3 interaction (Fig. 3C). Both CL and PA possess a high negative intrinsic curvature at neutral pHs^37^, and when incorporated to bilayers they could create membrane defects in turn helping ATG3 insertion. According to this, the electrostatic component would favor protein binding but it would not be the only force driving this association.

BAR domains and amphipathic α-helices are considered to be the best examples of membrane curvature-sensing domains in proteins. BAR domains sense membrane curvature by insertion of an amphipathic helix into membrane defects and also by electrostatic interactions with negative charges in the membrane^38^, whereas amphipathic α-helices such as ALPS motifs just insert their hydrophobic face into lipid packing defects^39,40^. ATG3 exhibits both capabilities, detecting electrostatics and intrinsic curvature of the membrane lipids. According to the “snorkel model”, lysines at the interface of the polar and nonpolar faces of the helix favor the association to flat membranes and the electrostatic interaction with them^41^, and this would also explain ATG3 ability to bind negatively-charged vesicles. Hence, mutations of lysine residues to aspartic acid in ATG3 K9D/K11D mutant would cause an electrostatic repulsion between these amino acids and the negative charges of the membrane (lipid polar head groups and phosphate). A comparable Lys/Ala mutation slightly decreases the binding to anionic vesicles but not to neutral membranes (data not shown). In the autophagy context, ATG3 would not be the only protein proposed to sense membrane curvature through an amphipathic helix, Barkor is also involved in detecting this curvature in phosphatidylinositol 3-phosphate (PI3P)-enriched membranes^42,43^.

Moreover, there are evidences suggesting that the autophagosome biogenesis is a lipid-modulated process^44^. While the role of phosphoinositides (PI3P, PI4P, PI5P, PI(4,5)P_2_) in autophagy progression is well known^44–46^, little information on the participation of other lipid species during the different steps of autophagosome biogenesis has been reported^47,48^. A combined action between lipids and autophagy proteins should lead to phagophore expansion. The participation of PA and other cone-shaped lipids (e.g. DAG) in autophagy and also in membrane fusion/fission processes has been proposed^49,50^. Previous studies from our group have suggested a possible role of intrinsic-curvature modifying lipids such as CL, DAG and LPC in promoting GATE-16 and GABARAP mediated vesicle fusion *in vitro* ^10^. DAG generation was shown to be required for the initiation of *Salmonella*-induced autophagy^51^. Moreover the PLD1 pathway, that produces PA, a cone-shaped lipid, has been also reported to promote autophagy^52–54^. The lipid composition of the pre-autophagosomal membrane is not yet known but its description would open a new window to understand how AP elongation would occur.

### ATG3 promotes vesicle aggregation and enhances LC3/GABARAP lipidation

During autophagosome formation and elongation, a large amount of vesicles containing protein and lipid material are fused with the nascent autophagosome. In this process some Atg proteins are involved, i.e the Atg7/Atg3/Atg8 UBL system. Atg8 homologues are the final effectors of the system and mediate the tethering and fusion of liposomes *in vitro* ^10^. It is also known that other proteins e.g. SNARES participate in this action^55^. Here we report a novel function for ATG3 as a tethering protein (Figs 4 and 6). The aggregation potential would be related to the anionic charge and intrinsic lipid curvature, but further work has to be performed to find the specific protein regions involved in this activity. In this context, ATG3 might be cooperating with Atg8 in the aggregation process prior to vesicle fusion in the pre-autophagosome elongation.

Other studies have reported a regulation of the lipidation ratio of Atg8 and their mammalian homologues through changes in the membrane lipid composition^4,10,15,35^. According to this, we suggest in this work that ATG3 would be responsible for bringing Atg8 homologues to the membrane. As we have shown, the *in vitro* incorporation of CL, PA or PI to liposomes improves ATG3 association to the membrane and also enhances the lipidation ratio of GABARAP (Fig. 8). Nevertheless, other proteins participate in this process *in vivo* such as the Atg12-Atg5-Atg16L1 system, and further experiments with the latter partners should be performed to discern the precise role of each protein.

In conclusion, the above data on ATG3 interaction with membranes should help clarify the mechanism by which the lipid composition of the autophagosomal membrane would facilitate redirecting the autophagy machinery to some regions, for the elongation process to occur. However, the phagophore lipid composition and the number of proteins recruited to it must be further investigated to understand the phagophore elongation process. This would lead to the discovery of new potential targets involved in macroautophagy that could also be explored in cancer progression therapies.

## Materials and Methods

### Materials

Egg phosphatidylcholine (PC) was purchased from Lipid Products (South Nutfield, UK). 1,2-dioleoyl-*sn*-glycero-3-phosphoethanolamine (DOPE), bovine liver phosphatidylinositol (PI), heart bovine cardiolipin (CL), brain phosphatidylserine (PS), egg phosphatidyldglycerol (PG), egg phosphatidic acid (PA), 1,2-dioleoyl-*sn*-glycero-3-phosphoethanolamine-N-(lissamine rhodamine B sulfonyl) (Rho-PE), ovine wool cholesterol (Chol) and 1,2-dihexadecanoyl-*sn*-glycero-3-phosphocholine (DPPC) were purchased from Avanti Polar Lipids, Inc. (Alabaster, AL). Anti-ATG3 antibody and goat anti-mouse IgG-HRP were from Santa Cruz Biotechnology, Inc. (Dallas, TX). Anti-GABARAP monoclonal antibody was from MBL International (Medical & Biological Laboratories Co. Ltd.). Alexa Fluor® 488 dye was purchased from Molecular Probes (Eugene, OR). All other reagents were of analytical grade.

### DNA constructs and site-directed mutagenesis

Plasmids for expression of human ATG3, ATG7 and GABARAP were kindly provided by Dr. Isei Tanida (National Institute of Infectious Diseases, Tokyo, Japan). A double mutant for Lys to Asp at positions 9 and 11 of ATG3 (ATG3 K9D/K11D) was constructed using a QuikChange site-directed mutagenesis kit (Stratagene, San Diego, California). Specifically designed ATG3 primers to introduce site-directed mutations were purchased from Sigma Aldrich (Madrid, Spain). The PCR-amplification products were evaluated by agarose gel electrophoresis and the parental methylated and hemimethylated DNA was digested by DpnI endonuclease (New England Biolabs). After inactivation of DpnI (80 °C for 20 min), the digested PCR product was transformed into DH5-α E. coli chemo-competent cells and inoculated on Luria–Bertani (LB) plates containing 100 mg/ml ampicillin. A total of 5 colonies were selected and their plasmids were isolated by mini-prep (GeneJET Plasmid Miniprep Kit, Thermo Scientific). Mutations were confirmed by DNA sequencing (Secugen S.L, Madrid, Spain).

### Recombinant protein expression and purification

ATG3 variants and GABARAP were purified from soluble fractions of bacterial extracts obtained in the absence of detergents, and were >90% pure as evaluated by Coomassie-stained SDS-PAGE. *E.coli* BL21 (λDE3) cells were transformed with appropriate plasmids, grown to OD_600_ = 0.8 and induced with 0.5 mM IPTG for 18 h at 20 °C. Following centrifugation at 4,500 xg for 15 min, the pellet was resuspended and sonicated in breaking buffer (PBS with protease inhibitors mixture and 1 mM DTT). After removal of cellular debris by centrifugation at 30,000 xg for 30 min at 4 °C, the sample supernatant fraction was incubated with 1 ml Glutathione Sepharose 4B (GE Healthcare) for 3 h at 4 °C to bind GST tagged proteins. Then, PreScission Protease (GE Healthcare) was added at 100 units/ml in 2 bed volumes of PreScission Buffer [50 mM Tris (pH 7.5), 150 mM NaCl, 1 mM EDTA] with freshly added 1 mM DTT and cleavage was performed for 4 h at 4 °C. Cleaved protein was eluted and purified proteins were stored in 20% glycerol at −80 °C.

ATG7 was expressed by baculoviral infection of HighFive (H5) insect cells. ATG7 in pFASTBAC HTa plasmid DNA was used to transform DH10Bac *E. coli* for transposition into the bacmid. We used blue/white colony selection to identify colonies containing the recombinant bacmid and they were confirmed by polymerase chain reaction. The recombinant bacmid was then isolated and purified using the Macherey Nagel endotoxin-free DNA purification kit. H5 insect cells were transfected with purified bacmid using Lipofectamine from Invitrogen (Waltham, MA) in TC-100 insect media (Sigma-Aldrich) supplemented with the appropriate antibiotics. When the transfected cells demonstrated signs of late stage infection (typically around 72 h), the medium containing the free virus was collected. We repeated cycles of transfection and virus collection to amplify the viral stock. Cells were collected after 48 h infection followed by centrifugation at 5000 xg for 10 min. The pellet was resuspended and sonicated in a breaking buffer consisting of 50 mM Tris pH 8, 150 mM NaCl, and freshly prepared 1 mM TCEP and protease inhibitors. The lysate was cleared by 30 min centrifugation at 30,000 xg and loaded on cobalt resin (Clontech, Mountain View, CA) for 3 h at 4 °C. Protein was eluted with 500 mM imidazole, concentrated up to 500 μl using YM-30 microcons (Millipore, Darmstadt, Germany) and loaded onto a Superdex TM200HR 10/30 size exclusion column (GE Healthcare, Buckinghamshire, UK) equilibrated in 50 mM Tris pH 8, 150 mM NaCl, supplemented with freshly added 1 mM DTT and protease inhibitors. Purified protein was kept at 4 °C.

### Monolayer surface pressure measurements

Lateral pressure experiments were carried out in a multi-well Delta Pi-4 Langmuir balance (Kibron Inc., Helsinki, Finland) under constant stirring. Lipid monolayers were formed by spreading a small amount of the lipid mixtures in chloroform:methanol (2:1 v/v) solution on top of assay buffer, until the desired initial surface pressure was reached. Proteins were injected with a micropipette through a hole connected to the subphase. Upon protein addition, changes induced in surface pressure at the air-water interface and protein-lipid monolayer interactions were studied at 25 °C (same results were obtained at 37 °C, data not shown). The assay buffer was 20 mM HEPES [4-(2-hydroxyethyl)−1-piperazineethanesulfonic acid] (pH 7.4), 150 mM NaCl, 1 mM MgCl_2_, 0.2 mM DTT.

### Liposome preparation

The appropriate lipids were mixed in organic solution and the solvent was evaporated to dryness under a N_2_ stream. Then the sample was kept under vacuum for 2 h to remove solvent traces. The lipids were swollen in the desired buffer in order to obtain multilamellar vesicles (MLVs). Large unilamellar vesicles (LUVs) were prepared from MLVs. They were subjected to 10 freeze/thaw cycles, then extruded using 0.1 µm pore size Nuclepore filters as described by Mayer *et al*.^56^. In the case of small unilamellar vesicles (SUVs), they were obtained by sonicating MLVs with a probe tip sonicator (MSE Soniprep 150 (MSE, UK)) for 10 min (10 s on, 10 s off) on ice. Vesicle size was checked by quasi-elastic light scattering using a Malvern Zeta-Sizer 4 spectrometer (Malvern Instruments, Worcestershire, UK). LUVs had an average diameter of ≈100 nm and SUVs an average diameter of ≈50 nm. Phospholipid concentration was determined with a phosphate assay^57^.

### Sucrose gradient centrifugation of liposomes

ATG3 purified as indicated (10 µM) was incubated with 3 mM liposomes (containing 0.05% Rho-PE for detection) for 1 h at 37 °C in 200 µl PreScission buffer. The protein/lipid mix was diluted to 300 µL in PreScission buffer containing 2.4 M sucrose. Then the reaction mix was transferred to a centrifuge tube, and the sample layer was overlaid with 400 µl PreScission buffer containing 0.8 M sucrose and subsequently 300 µL PreScission buffer containing 0.5 M sucrose. Sucrose step gradients were centrifuged in a TLA-120.2 rotor at 100,000 xg for 3 h at 4 °C. After that, four 250-µL fractions were pipetted starting from the bottom. The bottom fraction (B) contained the unbound protein, and the top fraction (T) contained liposomes as determined by the rhodamine fluorescence. Both fractions were analyzed by SDS-PAGE and Western blotting. The liposome-bound protein fraction was further quantified by densitometric integration of the electrophoretic peaks.

### Aggregation assays

Assays were carried out at 37 °C with continuous stirring, in a 1 mL cuvette with 600 μL of 50 mM Tris, 150 mM NaCl, 1 mM DTT buffer (pH 7.5). All experiments were performed at a vesicle concentration equivalent to 0.4 mM phosphate and 10 μM ATG3, unless otherwise stated. Lipid aggregation was monitored in a Uvikon 922 spectrophotometer (Kontron instruments, Groß-Zimmern, Germany) as an increase in turbidity (absorbance at 400 nm) of the sample.

### Alexa Fluor 488 protein labeling

Purified proteins were concentrated to 4–5 mg/mL and dialyzed against 0.1 M sodium bicarbonate pH 8.2, 150 mM NaCl buffer to remove any amine-containing substances that could interfere with the conjugation reaction. Then the protein solution was slowly mixed with 10 µl of the reactive dye solution (10 mg/mL of the amine-reactive compound dissolved in dimethylsulfoxide). The reaction mixture was incubated for 2 h at 37 °C with continuous stirring. Using a Sephadex G-25 chromatography column the conjugate was separated from unreacted labelling reagent, with PreScission buffer as the eluent. The degree of labelling was determined measuring the absorbance of the protein-dye conjugate at 280 nm and that of the dye at 488 nm.

### Giant Unilamellar Vesicle (GUV) preparation

GUVs were prepared using the electroformation method detailed below. For direct visualization under the microscope a homemade chamber was used^58^. Transferred GUVs were formed in a PRETGUV 4 chamber supplied by Industrias Técnicas ITC (Bilbao, Spain). Stock solutions of lipids (0.2 mM total lipid containing 0.2 mol% Rho-PE) were prepared in chloroform:methanol (2:1, v/v), 3 μL of the lipid stocks were added onto the surface of platinum (Pt) electrodes and solvent traces were removed by drying the chamber under high vacuum for at least 2 h.

### Direct visualization of GUVs

The Pt electrodes were covered with 400 μl of 50 mM Tris-HCl, 150 mM NaCl, 1 mM EDTA, 1 mM DTT, pH 7.5 buffer. The Pt wires were connected to an electric wave generator (TG330 function generator, Thurlby Thandar Instruments, Huntington, UK) under alternating current (AC) field conditions (500 Hz, 0.031 VRMS for 6 min; 500 Hz, 0.281 VRMS for 20 min, and 500 Hz, 0.623 VRMS for 1 h 30 min) at 37 °C. After GUV formation, the chamber was placed on an inverted confocal fluorescence microscope (Nikon D-ECLIPSE C1, Nikon, Melville, NY). The excitation wavelengths were 488 nm for ATG3-Alexa488 and 561 nm for Rho-PE. The images were collected using band-pass filters of 593 ± 20 nm for Rho-PE, and of 515 ± 15 nm for Alexa488. Then 1 μM ATG3-Alexa488 was added to study the ATG3 effect on the GUVs. All these experiments were performed at room temperature. Image treatment was performed using the EZ-C1 3.20 software (Nikon).

### Observation of transferred GUVs

The Pt electrodes were covered with 400 μl of a 300 mM sucrose solution, previously equilibrated at 37 °C. The Pt electrodes were connected to a generator (TG330 function generator, Thurlby Thandar Instruments) under AC field conditions (10 Hz, 1 VRMS for 2 h, followed by 2.5 Hz, 1 VRMS, 1 h 30 min) at 37 °C. Finally, the AC field was turned off and the vesicles (in 300 mM sucrose) were collected from the PRETGUV 4 chamber with a pipette and transferred to chambers pretreated with bovine serum albumin (BSA) (2 mg/ml) and containing an equiosmolar buffer solution of 50 mM Tris-HCl, 150 mM NaCl, 1 mM EDTA, 1 mM DTT, pH 7.5. Due to the different density of the two solutions, the vesicles sedimented at the bottom of the chamber, and this facilitated observation under the microscope. Finally, ATG3-Alexa488 at 1 μM was added to study the ATG3 effect on the GUVs. The excitation wavelengths were 488 nm for ATG3-Alexa488 and 561 nm for Rho-PE; and the emission was collected using 515 ± 15 nm and 593 ± 20 nm band-pass filters, respectively. All these experiments were performed at room temperature. Image treatment was performed using the EZ-C1 3.20 software (Nikon).

### Lipid nanotube formation

Lipid nanotubes (NTs) were formed by surface spreading of supported bilayers with excess membrane reservoir (SUPER templates^32^). Briefly, stock 100-nm LUVs containing egg PC:DOPE:Rho-PE, 69:30:1 mol% at 1 mM total lipid concentration were prepared in 20 mM Hepes pH 7.5, 150 mM NaCl. Stock LUVs were incubated with 40 μm-diameter silica beads as described earlier^33^ in order to obtain the SUPER templates. SUPER templates were then introduced into a homemade chamber made with two polydimethylsiloxane (PDMS) ribbons forming a thin (~1 mm high, 5 mm wide, 15 mm long) channel between a 25–35 mm round cover glass at the bottom and a 15 mm round cover glass at the top. The bottom cover glass was introduced into a coverslip dish (Harvard Apparatus, Holliston, Massachusetts), which was used for visualization of the sample on an inverted confocal microscope (Leica TCS-SP5). A small aliquot containing the SUPER templates was introduced from one side (loading side) of the channel previously filled up with the working buffer. SUPER templates were allowed to settle down for 5 minutes, and then the chamber was carefully tilted 45–65 degrees with the loading side heading up. The cloud of SUPER templates was observed to descend the channel for 3/4 of its length, allowing for NT formation between the beads, and on the lower coverslip surface. To decrease fluorescent background, only the NTs formed between the beads were observed. Upon NT formation, ATG3-containing solution was introduced from the loading side and was allowed to diffuse for 30 minutes along the channel, thus avoiding physical perturbation of the NTs. The ATG3 final concentration in the experimental channel was 1 μM.

## Electronic supplementary material

Supplementary Information

## References

[1] Tooze Sa (2010). Trafficking and signaling in mammalian autophagy. IUBMB Life.

[2] He C, Klionsky DJ (2009). Regulation Mechanisms and Signaling Pathways of Autophagy. Annu. Rev. Genet..

[3] Lamb Ca, Yoshimori T, Tooze Sa (2013). The autophagosome: origins unknown, biogenesis complex. Nat. Rev. Mol. Cell Biol..

[4] Sou Y-s, Tanida I, Komatsu M, Ueno T, Kominami E (2006). Phosphatidylserine in Addition to Phosphatidylethanolamine Is an *in Vitro* Target of the Mammalian Atg8 Modifiers, LC3, GABARAP, and GATE-16. J. Biol. Chem..

[5] Tanida I, Ueno T, Kominami E (2004). LC3 conjugation system in mammalian autophagy. Int. J. Biochem. Cell Biol..

[6] Kaiser SE (2012). Noncanonical E2 recruitment by the autophagy E1 revealed by Atg7–Atg3 and Atg7–Atg10 structures. Nat. Struct. Mol. Biol..

[7] Hong SB (2011). Insights into noncanonical E1 enzyme activation from the structure of autophagic E1 Atg7 with Atg8. Nat. Struct. Mol. Biol..

[8] Weidberg H (2011). LC3 and GATE-16 N Termini Mediate Membrane Fusion Processes Required for Autophagosome Biogenesis. Dev. Cell.

[9] Nakatogawa H, Ichimura Y, Ohsumi Y (2007). Atg8, a ubiquitin-like protein required for autophagosome formation, mediates membrane tethering and hemifusion. Cell.

[10] Landajuela A (2016). Lipid geometry and bilayer curvature modultae LC3/GABARAP-mediated model autophagosome elongation. Biophys. J..

[11] Popelka H, Uversky VN, Klionsky DJ (2014). Identification of Atg3 as an intrinsically disordered polypeptide yields insights into the molecular dynamics of autophagy-related proteins in yeast. Autophagy.

[12] Yamada Y (2007). The Crystal Structure of Atg3, an Autophagy-related Ubiquitin Carrier Protein (E2) Enzyme that Mediates Atg8 Lipidation. J. Biol. Chem..

[13] Sakoh-Nakatogawa M (2013). Atg12–Atg5 conjugate enhances E2 activity of Atg3 by rearranging its catalytic site. Nat. Struct. Mol. Biol..

[14] Hanada T, Satomi Y, Takao T, Ohsumi Y (2009). The amino-terminal region of Atg3 is essential for association with phosphatidylethanolamine in Atg8 lipidation. FEBS Lett..

[15] Nath S (2014). Lipidation of the LC3/GABARAP family of autophagy proteins relies on a membrane-curvature-sensing domain in Atg3. Nat. Cell Biol..

[16] Li Y-T (2017). A semisynthetic Atg3 reveals that acetylation promotes Atg3 membrane binding and Atg8 lipidation. Nat. Commun..

[17] Radoshevich L (2010). ATG12 Conjugation to ATG3 Regulates Mitochondrial Homeostasis and Cell Death. Cell.

[18] Metlagel Z, Otomo C, Takaesu G, Otomo T (2013). Structural basis of ATG3 recognition by the autophagic ubiquitin-like protein ATG12. Proc. Natl. Acad. Sci. USA.

[19] Romanov J (2012). Mechanism and functions of membrane binding by the Atg5–Atg12/Atg16 complex during autophagosome formation. EMBO J..

[20] Walczak M, Martens S (2013). Dissecting the role of the Atg12-Atg5-Atg16 complex during autophagosome formation. Autophagy.

[21] Klionsky DJ, Schulman Ba (2014). Dynamic regulation of macroautophagy by distinctive ubiquitin-like proteins. Nat. Struct. Mol. Biol..

[22] Liu K (2016). ATG3-dependent autophagy mediates mitochondrial homeostasis in pluripotency acquirement and maintenance. Autophagy.

[23] Marsh D (1996). Lateral pressure in membranes. Biochim. Biophys. Acta - Rev. Biomembr..

[24] Nair U (2011). SNARE proteins are required for macroautophagy. Cell.

[25] Israelachvili J, Marcelja S, Horn R (1980). Physical principles of membrane organization. Q. Rev. ….

[26] Alonso A, Goñi FM, Buckley JT (2000). Lipids favoring inverted phase enhance the ability of aerolysin to permeabilize liposome bilayers. Biochemistry.

[27] Martín C (2004). Membrane restructuring by Bordetella pertussis adenylate cyclase toxin, a member of the RTX toxin family. J. Bacteriol..

[28] Jamil H, Hatch GM, Vance DE (1993). Evidence that binding of CTP:phosphocholine cytidylyltransferase to membranes in rat hepatocytes is modulated by the ratio of bilayer- to non-bilayer-forming lipids. Biochem. J..

[29] Goñi FM, Villar AV, Nieva JL, Alonso A (2003). Interaction of phospholipases C and sphingomyelinase with liposomes. Methods Enzymol..

[30] Veatch SL, Keller SL (2003). Separation of liquid phases in giant vesicles of ternary mixtures of phospholipids and cholesterol. Biophys. J..

[31] Beales PA, Bergstrom CL, Geerts N, Groves JT, Vanderlick TK (2011). Single vesicle observations of the cardiolipin-cytochrome C interaction: induction of membrane morphology changes. Langmuir.

[32] Neumann S, Pucadyil TJ, Schmid SL (2013). Analyzing membrane remodeling and fission using supported bilayers with excess membrane reservoir. Nat. Protoc..

[33] Pucadyil TJ, Schmid SL (2010). Supported Bilayers with Excess Membrane Reservoir: A Template for Reconstituting Membrane Budding and Fission. Biophys. J..

[34] Hanada T (2007). The Atg12-Atg5 conjugate has a novel E3-like activity for protein lipidation in autophagy. J. Biol. Chem..

[35] Ichimura Y (2004). *In vivo* and *in vitro* reconstitution of Atg8 conjugation essential for autophagy. J. Biol. Chem..

[36] Oh-oka K, Nakatogawa H, Ohsumi Y (2008). Physiological pH and Acidic Phospholipids Contribute to Substrate Specificity in Lipidation of Atg8. J. Biol. Chem..

[37] Frolov VA, Shnyrova AV, Zimmerberg J (2011). Lipid polymorphisms and membrane shape. Cold Spring Harb. Perspect. Biol..

[38] Madsen KL, Bhatia VK, Gether U, Stamou D (2010). BAR domains, amphipathic helices and membrane-anchored proteins use the same mechanism to sense membrane curvature. FEBS Lett..

[39] Bigay J, Casella J-F, Drin G, Mesmin B, Antonny B (2005). ArfGAP1 responds to membrane curvature through the folding of a lipid packing sensor motif. EMBO J..

[40] Mesmin B (2007). Two lipid-packing sensor motifs contribute to the sensitivity of ArfGAP1 to membrane curvature. Biochemistry.

[41] Mishra VK, Palgunachari MN, Segrest JP, Anantharamaiah GM (1994). Interactions of synthetic peptide analogs of the class A amphipathic helix with lipids. Evidence for the snorkel hypothesis. J. Biol. Chem..

[42] Fan W, Nassiri a, Zhong Q (2011). Autophagosome targeting and membrane curvature sensing by Barkor/Atg14(L). Proc. Natl. Acad. Sci..

[43] Wilz L, Fan W, Zhong Q (2011). Membrane curvature response in autophagy. Autophagy.

[44] Dall’Armi C, Devereaux KA, Di Paolo G (2013). The Role of Lipids in the Control of Autophagy. Curr. Biol..

[45] Dooley HC (2014). WIPI2 Links LC3 Conjugation with PI3P, Autophagosome Formation, and Pathogen Clearance by Recruiting Atg12–5-16L1. Mol. Cell.

[46] Vicinanza M (2015). PI(5)P regulates autophagosome biogenesis. Mol. Cell.

[47] Sentelle RD (2012). Ceramide targets autophagosomes to mitochondria and induces lethal mitophagy. Nat. Chem. Biol..

[48] Chu CT (2013). Cardiolipin externalization to the outer mitochondrial membrane acts as an elimination signal for mitophagy in neuronal cells. Nat. Cell Biol..

[49] Ibarguren M (2010). End-products diacylglycerol and ceramide modulate membrane fusion induced by a phospholipase C/sphingomyelinase from Pseudomonas aeruginosa. Biochim. Biophys. Acta.

[50] Nieva JL (1995). Topological properties of two cubic phases of a phospholipid: cholesterol: diacylglycerol aqueous system and their possible implications in the phospholipase C-induced liposome fusion. FEBS Lett..

[51] Shahnazari S, Namolovan A, Klionsky DJ, Brumell JH (2011). A role for diacylglycerol in antibacterial autophagy. Autophagy.

[52] Dall’Armi C (2010). The phospholipase D1 pathway modulates macroautophagy. Nat. Commun..

[53] Jenkins GM, Frohman MA (2005). Phospholipase D: a lipid centric review. Cell. Mol. Life Sci..

[54] Foster Da (2007). Regulation of mTOR by Phosphatidic Acid?. Cancer Res..

[55] Moreau K, Renna M, Rubinsztein DC (2013). Connections between SNAREs and autophagy. Trends Biochem. Sci..

[56] Mayer LD, Hope MJ, Cullis PR (1986). Vesicles of variable sizes produced by a rapid extrusion procedure. Biochim. Biophys. Acta - Biomembr..

[57] Böttcher CJF, Van gent CM, Pries C (1961). A rapid and sensitive sub-micro phosphorus determination. Anal. Chim. Acta.

[58] Fidorra M, Duelund L, Leidy C, Simonsen AC, Bagatolli LA (2006). Absence of fluid-ordered/fluid-disordered phase coexistence in ceramide/POPC mixtures containing cholesterol. Biophys. J..

[59] Ulmschneider MB (2014). Spontaneous transmembrane helix insertion thermodynamically mimics translocon-guided insertion. Nat. Commun..

