# Supplementary material for: Human ATG3 binding to lipid bilayers: role of lipid geometry, and electric charge
Source: Sci Rep. 2017 Nov 15;7:15614. doi: 10.1038/s41598-017-15057-6 (PMC5688168; doi:10.1038/s41598-017-15057-6)

Supplementary Information

Human ATG3 binding to lipid bilayers: role of lipid geometry, and electric charge

Javier H. Hervás, Ane Landajuela, Zuriñe Antón, Anna V. Shnyrova, Felix M. Goñi and Alicia Alonso*

Instituto Biofisika (CSIC, UPV/EHU) and Departamento de Bioquímica y Biología Molecular, Universidad del País Vasco, Bilbao, Spain.

*Corresponding author. Email: alicia.alonso@ehu.eus

Supplementary Figure 1

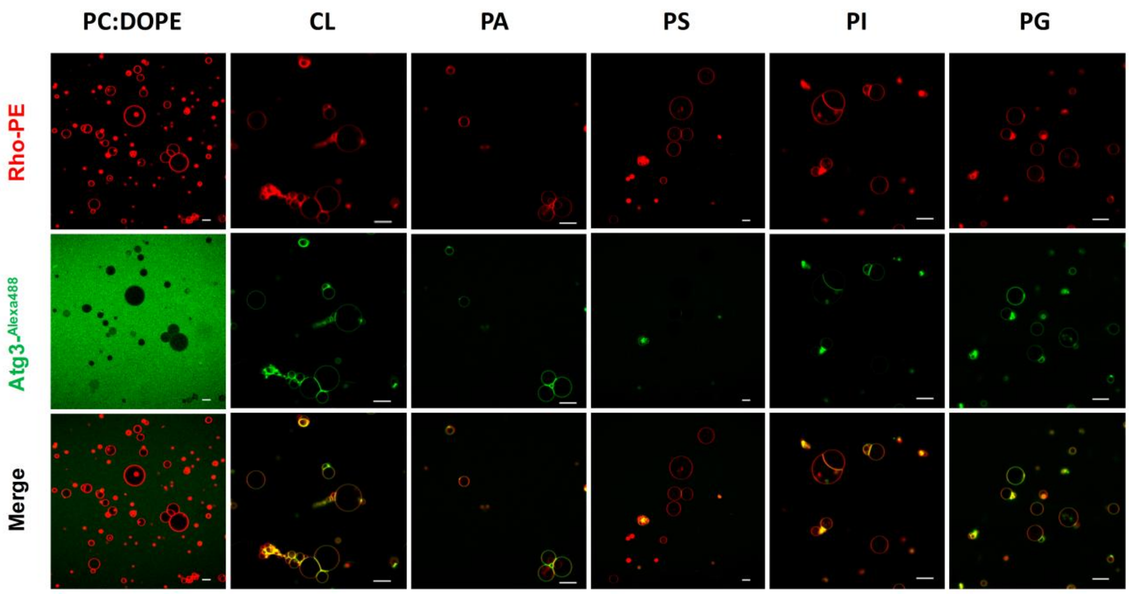

Figure S1. ATG3 interaction with negatively-charged GUVs. Different anionic phospholipids are incorporated to GUVs (labeled with Rhodamine-PE) and protein (labeled with Alexa-488) incorporation to them was observed by confocal microscopy. Merge: yellow indicates colocalization of both probes. Scale bars: 10 μm.

Supplementary Figure 2

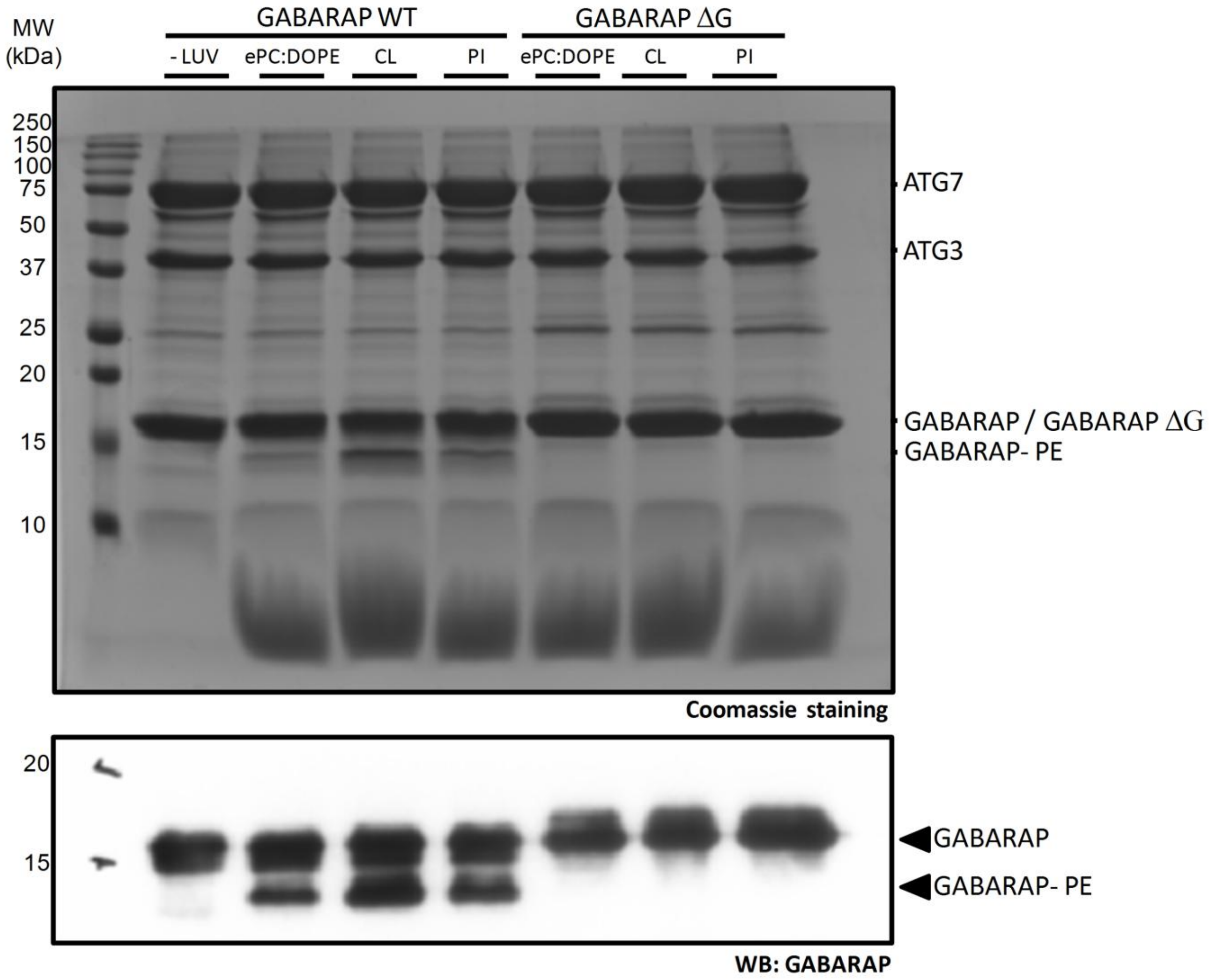

Figure S2. Coomassie blue staining (upper panel) and anti-GABARAP Western blotting (lower panel) of the *in vitro* lipidation reaction using different lipid compositions. Lipid compositions are ePC:DOPE (70:30 mol ratio), ePC:DOPE:CL (60:30:10 mol ratio) and ePC:DOPE:PI (50:30:20 mol ratio). Black arrow heads indicate lipidated and non-lipidated forms of GABARAP.

Supplementary Figure 3

Figure 3 uncropped gels

Figure 3A (αATG3 western Blot)

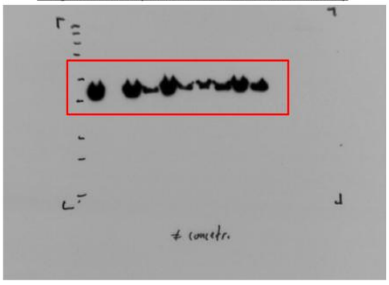

Figure 3B (αATG3 western Blot)

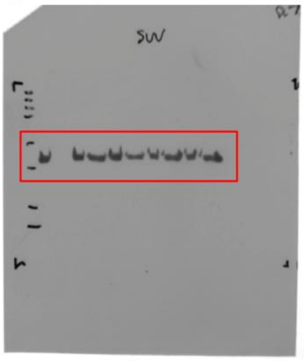

Figure 3C (αATG3 western Blot)

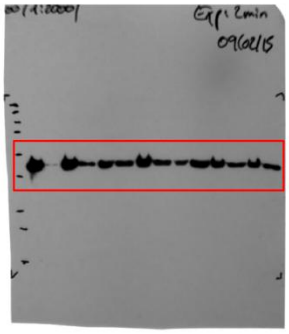

Figure 8 uncropped gels

Figure 8D (coomassie)

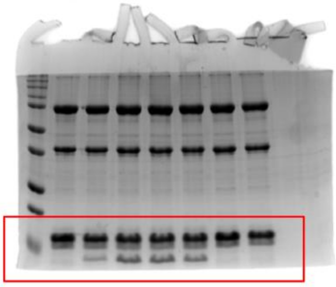

Figure 8D (αGABARAP western Blot)

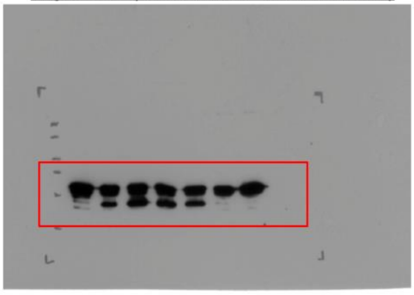

Supplement: Supplementary file 1 — Supplementary Information [file 41598_2017_15057_MOESM1_ESM.pdf]
